# Micro-endoscopic retrograde cholangiopancreatography catheter-assisted endoscopic laser lithotripsy: improving insertability and safety

**DOI:** 10.1055/a-2705-2491

**Published:** 2025-10-02

**Authors:** Takeshi Ogura, Junichi Nakamura, Takafumi Kanadani, Kimi Bessho, Hiroki Nishikawa

**Affiliations:** 1 13010 Pancreatobiliary Advanced Medical Center, Osaka Medical and Pharmaceutical University, Osaka, Japan; 2 13010 Endoscopy Center, Osaka Medical and Pharmaceutical University, Osaka, Japan; 3 13010 2nd Department of Internal Medicine, Osaka Medical and Pharmaceutical University, Osaka, Japan

Endoscopic laser lithotripsy (ELL) can be indicated for stone fragmentation or tumor ablation 1 2 3 . A 9-Fr pancreatocholangioscope (eyeMAX, Micro-Tech, Nanjing, China) has recently become available. Although operability and insertion into the target site are easy owing to the slimness of this new scope, the working channel is also small, meaning insertion of accessory devices into the scope may be challenging. In particular, owing to the high rigidity of the ELL probe, this may become lodged within the channel if the scope is significantly angulated, making it impossible to advance the probe through the thin endoscope. To overcome this limitation, we recently performed ELL assisted by a micro-ERCP catheter (3 Fr; Damon ERCP catheter, HANACO Medical Co., Ltd., Saitama, Japan) ( Fig. 1 ). Herein we describe some technical tips for ERCP catheter-assisted ELL.

**Fig. 1:**
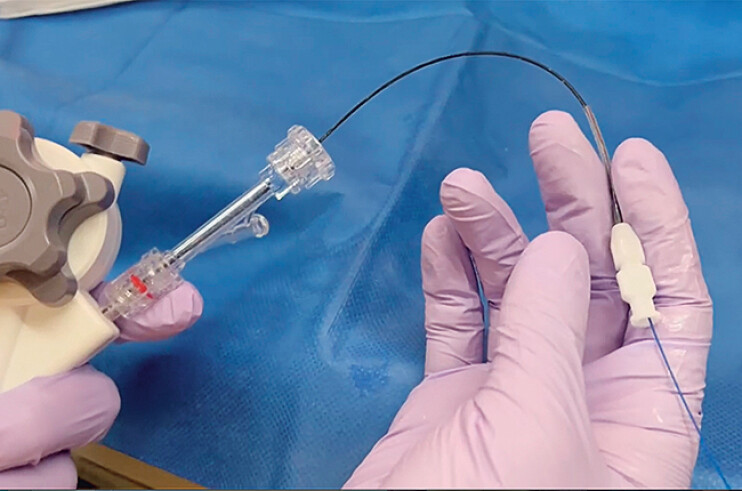
Photograph of the micro-endoscopic retrograde cholangiopancreatography (ERCP) catheter (3 Fr; Damon ERCP catheter, HANACO Medical Co., Ltd.).

A 71-year-old man was admitted to another hospital because of cholangitis due to primary sclerosing cholangitis. On cholangiography, right and left hepatic bile duct stenoses were observed; however, drainage had failed owing to obstruction of the left hepatic bile duct by a stone. Plastic stent deployment was performed for the right hepatic bile duct and he was then admitted to our hospital for drainage of the left hepatic bile duct.

First, biliary cannulation was performed and contrast medium was injected. An attempt was made to advance a 0.025-inch guidewire into the left hepatic bile duct, but this failed owing to the impacted bile duct stone ( Fig. 2 **a** ). The cholangioscope was then inserted into the left hepatic bile duct, and the stone was identified. ELL was attempted but, because the cholangioscope angle was acute, advancement of the probe through the scope to reach the stone was not possible ( Fig. 2 **b** ). Therefore, the micro-ERCP catheter was inserted and the probe was then inserted within this catheter. This allowed probe advancement to be easily and smoothly performed ( Fig. 3 ). In addition, the sheathed probe itself could be safely inserted without penetrating the bile duct. After stone fragmentation, guidewire advancement into the left intrahepatic bile duct was successfully performed. Finally, a plastic stent was deployed without any adverse events ( Fig. 4 ; Video 1 ).

**Fig. 2:**
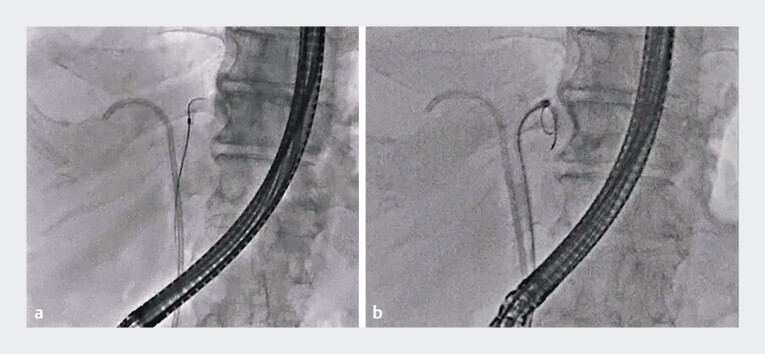
Fluoroscopic images showing: **a** failed insertion of a 0.025-inch guidewire into the left hepatic bile duct owing to an impacted bile duct stone; **b** failed advancement of the lithotripsy probe owing to the acute angulation of the cholangioscope.

**Fig. 3:**
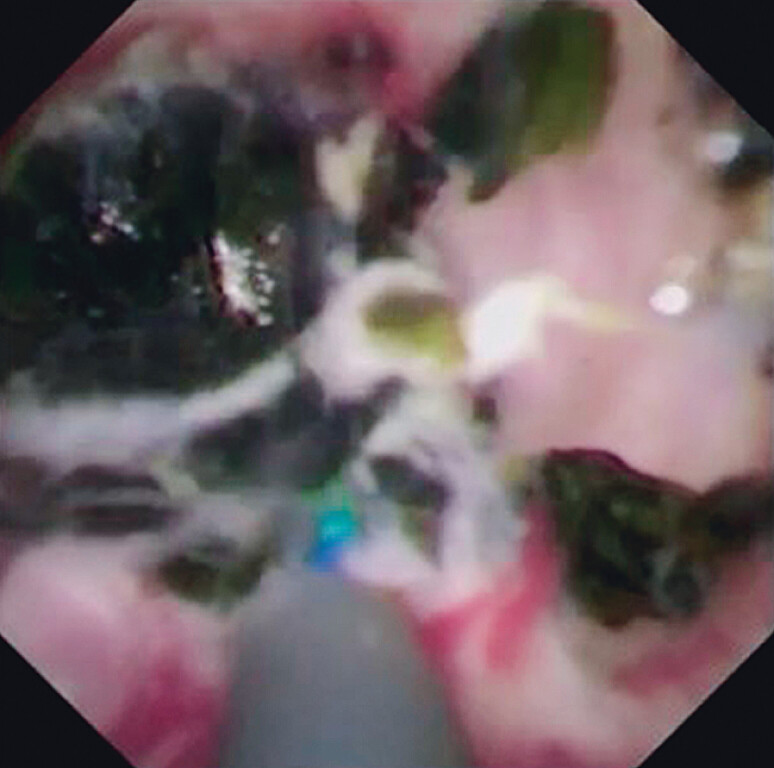
Cholangioscopic image showing the inserted micro-endoscopic retrograde cholangiopancreatography catheter through which the probe was then inserted, with easy and smooth advancement.

**Fig. 4:**
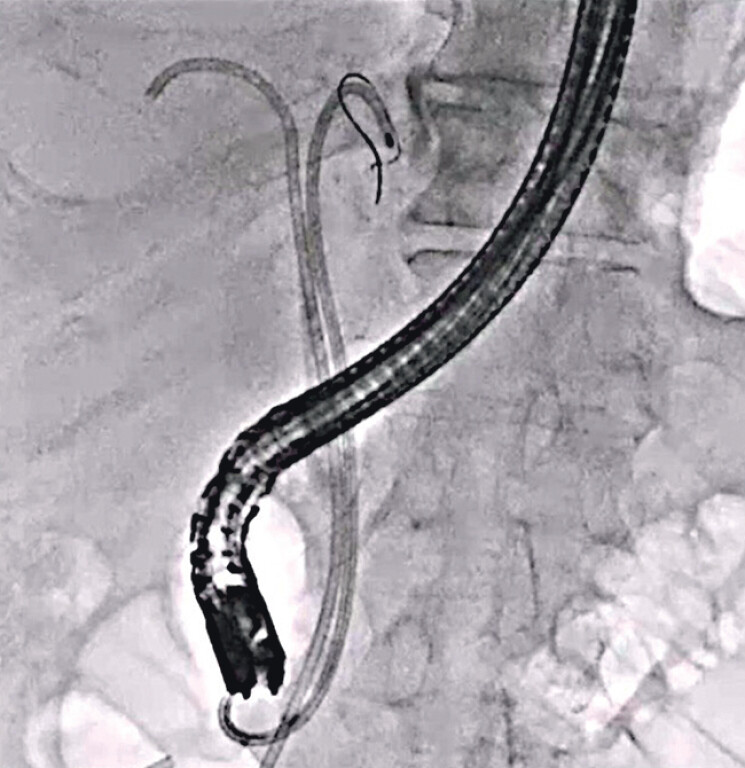
Fluoroscopic image showing plastic stent deployment.

Micro-endoscopic retrograde cholangiopancreatography catheter-assisted endoscopic laser lithotripsy is performed for an obstructing bile duct stone in the left hepatic duct.Video 1

In conclusion, micro-ERCP catheter-assisted ELL may be useful owing to the improved insertability and safety it offers.

Endoscopy_UCTN_Code_TTT_1AR_2AH

## Notes

Endoscopy E-Videos https://eref.thieme.de/e-videos
*E-Videos* is an open access online section of the journal *Endoscopy* , reporting on interesting cases and new techniques in gastroenterological endoscopy. All papers include a high-quality video and are published with a Creative Commons CC-BY license. Endoscopy E-Videos qualify for HINARI discounts and waivers and eligibility is automatically checked during the submission process. We grant 100% waivers to articles whose corresponding authors are based in Group A countries and 50% waivers to those who are based in Group B countries as classified by Research4Life (see: https://www.research4life.org/access/eligibility/ ). This section has its own submission website at https://mc.manuscriptcentral.com/e-videos .
